# LENGTH OF STAY IN REHABILITATION HOSPITALS AS WELL AS HOSPITAL ADMISSION IS ASSOCIATED WITH SACRAL ULCER DEVELOPMENT

**DOI:** 10.1093/geroni/igae098.1212

**Published:** 2024-12-31

**Authors:** Cheng Yin, Elias Mpofu, Kaye Brock

**Affiliations:** University of North Texas, Denton, Texas, United States; University of North Texas, Denton, Texas, United States; University of North Texas, Denton, Texas, United States

## Abstract

**Introduction:**

Sacral ulcers (SUs) pose a significant risk of mortality for older adults when admitted to rehabilitation hospitals. Previous research has underscored the influence of various factors including comorbidities, lifestyle, and personal factors on sacral ulcer development (SUD) in these settings. However, the impact of hospital admission and the mediating role of length of stay (LoS) on SUD remains unclear. Thus, this study aimed to determine the association between hospital admission and SUD in older adult patients in rehabilitation hospitals, and whether LoS mediated this association.

**Methods:**

Cross-sectional data were obtained from the Texas Inpatient Discharge Database (2021; N = 38,916 patients aged over 60 years in rehabilitation hospitals). Binary logistic regression was used to determine the association between hospital admission and SUD, mediation was then investigated.

**Results:**

Hospital admission increased the likelihood of SUD through mediation by LoS (b=0.30, 95% CI 0.26, 0.35). Accounting for covariates revealed hospital admission was associated with 1.58 times the odds of SUD (AOR = 1.58; 95% CI = 1.38-1.81), while LoS was linked to 1.06 times the odds of SUD (AOR = 1.06; 95% CI = 1.05-1.06).

**Conclusion:**

Our study extends previous findings, demonstrating hospital admission’s connection to increased odds of SUD, through prolonged LoS in rehabilitation hospitals. The results highlight the importance of categorizing the source of admission in vulnerable populations to mitigate potential risks of hospital-acquired infections and excessive mortality associated with SUs.

